# HLA-DRB1*11 and variants of the MHC class II locus are strong risk factors for systemic juvenile idiopathic arthritis

**DOI:** 10.1186/1546-0096-13-S1-O75

**Published:** 2015-09-28

**Authors:** M Ombrello, E Remmers, E Zeggini, W Thomson, D Kastner, P Woo

**Affiliations:** 1 NIAMS/NIH, Translational Genetics and Genomics Unit, Bethesda, USA; 2 NHGRI/NIH, Inflammatory Disease Section, Bethesda, USA; 3 Wellcome Trust Sanger Institute, Analytical Genomics of Complex Traits Group, Hinxton, UK; 4 University of Manchester, Arthritis Research UK Centre for Genetics and Genomics, Manchester, UK; 5 University College London, Division of Infection & Immunity, London, UK; 6 International Childhood Arthritis Genetics Consortium, London, UK

## Introduction

Systemic juvenile idiopathic arthritis (sJIA) is a severe, potentially life-threatening childhood inflammatory disease whose pathophysiology is poorly understood.

## Objective

To determine whether genetic variation of the major histocompatibility complex (MHC) locus influences sJIA susceptibility.

## Patients and methods

Single nucleotide polymorphism (SNP) genotypes were determined in 982 children with sJIA and 431 healthy subjects, and were combined with *in silico* SNP data from 7579 additional healthy subjects. The collection was divided into 9 strata by country of origin and subjected to stringent quality control procedures. MHC region SNPs and classical human leukocyte antigen (HLA) alleles and amino acids were determined by imputation and were tested for association with sJIA in the individual strata and by meta-analysis. To assess whether disease-associated MHC variants influenced sJIA through regulatory mechanisms, RegulomeDB was used to cross reference sJIA-associated SNPs with regulatory information from over 100 tissues and cell lines, including data from the ENCODE and NIH Roadmap Epigenome Projects.

## Results

Association testing and meta-analysis of MHC region SNPs in 9 strata found a strong, dominant association between sJIA and a 400 kb region of the MHC locus that included most of the class II HLA region. The strongest sJIA-associated SNP was rs151043342 (p=2.8E-17, OR 2.6 [2.1, 3.3]), and conditional analysis controlling for its effect revealed that a second SNP, rs12722051, independently influenced sJIA risk (p=1.0E-5, OR 0.7 [0.5, 0.8]). Meta-analysis of classical HLA type associations in the 6 strata of Western European ancestry revealed a strong association between sJIA and HLA-DRB1*11 (p=2.7E-16, OR 2.3 [1.9, 2.9]) and the HLA-DRB1*11-HLA-DQA1*05-HLA-DQB1*03 haplotype (p=6.4E-17, OR 2.3 [1.9, 2.9]). Examination of sJIA-associated SNPs (p<1.0E-5) with RegulomeDB identified 18 SNPs that were linked to expression of one or more class II HLA genes AND had strong evidence supporting an effect on transcription factor binding (RegulomeDB scores 1a - 1f). Importantly, the majority of sJIA-associated SNPs in the class II HLA locus intersected with super-enhancers that were cell-type specific for B cells and monocytes (as reported by Hnisz *et al.*, Cell 2013).

## Conclusions

Using meta-analysis of directly observed and imputed SNP genotypes and imputed classical HLA types, we identified the MHC locus as a *bona fide* sJIA susceptibility locus that influences disease risk in multiple independent populations. These data suggest that the class II HLA locus influences sJIA susceptibility through both protein coding variation and noncoding variation that alters gene expression.

